# Research progress of intra-aortic balloon counterpulsation in the treatment of acute myocardial infarction with cardiogenic shock: A review

**DOI:** 10.1097/MD.0000000000036500

**Published:** 2023-12-08

**Authors:** Mengxian Li, Liqun Hu, Lei Li

**Affiliations:** a Wuhan Puai Hospital Affiliated to Tongji Medical College of Huazhong University of Science and Technology, Cardiology Department, Qiaokou District, Wuhan City, Hubei Province, China; b Wuhan Fourth Hospital, Cardiology Department, Qiaokou District, Wuhan City, Hubei Province, China; c Wuhan Fourth Hospital, Cardiology Department, Qiaokou District, Wuhan City, Hubei Province, China.

**Keywords:** acute myocardial infarction, cardiogenic shock, intra-aortic balloon counterpulsation

## Abstract

The mortality rate of patients with acute myocardial infarction complicated with cardiogenic shock is very high, and in recent years, intra-aortic balloon counterpulsation has been used more and more. It plays a very important role in improving left ventricular ejection, increasing coronary artery perfusion pressure and reducing myocardial oxygen consumption. This article reviews the development of intra-aortic balloon counterpulsation in the treatment of acute myocardial infarction with cardiogenic shock in recent years.

## 1. Introduction

Cardiogenic shock is a state of low cardiac output, resulting in life-threatening terminal organ hypoperfusion and hypoxia. Acute myocardial infarction (AMI) complicated with left ventricular dysfunction is still the most common cause of cardiogenic shock. Although the survival rate has been improved in recent years, the morbidity and mortality of patients are still very high, cardiogenic shock is the main cause of death during hospitalization in patients with AMI.^[1]^ The global registration study shows that the overall incidence of AMI complicated with cardiogenic shock is 4%~12% and the 30-day mortality rate is as high as 40%~45%.^[2]^ Before routine application of early revascularization, the in-hospital mortality rate of cardiogenic shock is as high as 80%.^[3]^ Intra-aortic balloon counterpulsation (IABP) is the most widely used mechanical circulatory support device in cardiogenic shock,^[4]^ which provides short-term hemodynamic and clinical improvement for most patients.^[5]^ Although patients have received IABP treatment, the mortality rate is still high, according to the admission parameters, patients with a higher risk of death should be further treated with other mechanical circulatory support devices.^[6]^

## 2. Overview of IABP

### 2.1. Historical evolution and development

IABP is the most common mechanical support mode in heart failure. The increase of diastolic blood pressure during balloon inflation helps to improve the blood flow in coronary circulation, while balloon contraction can reduce the afterload and left ventricular ejection resistance, thus reducing myocardial work. The overall effect is to increase the ratio of myocardial oxygen supply and demand, thus increasing the endocardial survival rate. Moulopoulos and colleagues conducted a preliminary study of IABP in 1961 and tested their balloon pump in the simulated aorta of dogs and concluded that it increased diastolic blood flow in the arterial system and decreased end-diastolic arterial pressure.^[7]^ The concept of external counterpulsation was put forward by Dennis et al in 1963. They used a diastolic leg compression device to cover the lower extremities to the middle abdomen, inflated sequentially during diastole, and deflated synchronously with the contraction of the heart during systole. Finally, they found that it was feasible.^[8]^ In 1967, IABP was successfully used for the first time in a 45-year-old patient with myocardial infarction with cardiogenic shock, accompanied by hypotension, coma and anuria. By the early 1970s, the Kantrowitz team had successfully treated 30 patients, after that, IABP has been considered by many experts and scholars to be helpful in the treatment of acute low cardiac output after left heart failure. Buckley et al later studied the hemodynamic benefits of IABP and reported the results of treatment in 8 patients with cardiogenic shock. It was finally confirmed that diastolic balloon inflation can increase coronary perfusion pressure, reduce cardiac ejection resistance, and reduce cardiac work and oxygen consumption.^[9]^ Mundth and his colleagues reported a case of cardiogenic shock after myocardial infarction in 1970, which was stabilized by IABP, then underwent coronary artery revascularization and recovered successfully with balloon pump support.^[10]^ Jacobey et al reported the beneficial results of 18 patients with cardiogenic shock treated with ascending aortic catheterization in 1971.^[11]^ In 1971, Feola and colleagues observed the therapeutic effect of IABP on animal models of heart failure and concluded that IABP is an effective circulatory support method, provided it is used early and is most effective in mild to moderate left heart failure.^[12]^ In early 1971, Krakauer et al reported the experience of intra-aortic balloon therapy in 30 patients with AMI with cardiogenic shock who were ineffective to conventional drug therapy. They pointed out that counterpulsation can effectively affect hemodynamics and reduce ventricular wall end-diastolic pressure, thereby reducing myocardial tension and myocardial oxygen consumption. The increase of coronary perfusion pressure, coupled with the decrease of myocardial tension and the shortening of systolic ejection time, increased coronary blood flow. They concluded that patients who received balloon support early after shock had a significantly better prognosis.^[13]^ In 1972, Bregman and his colleagues developed a double-lumen airbag consisting of a large proximal balloon and a small distal balloon that inflated first. The idea was to stop the distal blood flow and increase blood flow to the brain and the proximal coronary artery. Since then, the development of polyurethane catheters and airbags has made long-term counterpulsation possible.^[14]^ In 1973, 2 different groups reported the successful use of IABP in patients who could not be separated from cardiopulmonary bypass. Since then, IABP support has ushered in a new era of perioperative management of patients with cardiac insufficiency during cardiac surgery.^[15,16]^ Continuous development has led to a method of percutaneous balloon catheterization without the need for surgery,^[17,18]^ which has changed the entire IABP field, as indications for the use of this device can be extended to different subgroups of patients with advanced coronary artery disease who are medically ineffective. With the continuous development of mechanical engineering technology, the best time to apply counterpulsation and the on-line monitoring of blood pressure and cardiac output become possible. since then, IABP has been widely recognized and developed rapidly in the cardiovascular field.^[19]^

### 2.2. Application indication

After full replenishment of blood volume, cardiac index (CI) < 2.0 L ·min-1 ·m-2; left ventricular ejection fraction < 30%; after cardiotonic treatment, mean arterial pressure < 50 mm Hg; urine volume < 20 mL/h. With high dose of dopamine ≥ 15 μg ·min-1 kg-1 and dobutamine ≥ 20 μg ·min-1 kg-1, the blood pressure still decreased; right atrial pressure (RAP) > 20 mmHg, left atrial pressure (LAP) > 20 mmHg; end circulation deficiency and cold at the end of the limb; low spirit, lack of tissue oxygen supply and low arteriovenous oxygen saturation.

### 2.3. Complications

According to various reports, left ventricular dysfunction, history of myocardial infarction, female, diabetes, peripheral vascular disease and left main artery disease are risk factors for increasing adverse reactions to IABP use.^[19]^ Common complications include lower limb ischemia, hemorrhage, thromboembolism, wound infection, anemia, arterial wall injury and dissection, balloon clamp, catheter failure to inflate or deflate effectively, and so on. In 1 study, 75 IABP patients had 5.3 per cent operative mortality and 6.6 per cent perioperative cerebral infarction.^[20]^ The complications of IABP are due to the difficulty of implantation, dislocation, long stay of IABP, peripheral angiopathy, small size, use of IABP sheath and diabetes. A paper by Rastan et al pointed out that IABP dislocation is a common finding in post-implantation CT scans. 68.2% of the cases found that the anatomical length did not match the balloon length, followed by serious adverse reactions.^[21]^ In addition, due to the nature of IABP, the main complications are related to vascular injury. Studies have shown that vascular ischemic complications occur in 8% to 18% of cases, while <1% of major limb ischemia is reported.^[22–26]^ The pulse of the foot disappeared in 29.5% of the cases. The ischemia disappeared after the balloon was removed or the thrombus was removed. One patient developed gangrene and required amputation.^[27]^ 57.9% of the patients had thrombocytopenia, defined as < 150,000/mL or > 50% below the baseline. Thrombocytopenia is usually mild and does not seem to be associated with simultaneous use of heparin or an increased risk of massive hemorrhage or hospital death.^[28,29]^ Rupture of IABP is rare, but can lead to gas embolism or balloon trapping in the artery. This was first reported by Rajani et al, but this situation is very rare and may be <0.5%.^[30]^ Complications of thrombosis and infection are related to the duration of IABP treatment, while limb ischemia problems depend more on the atherosclerotic state of the common femoral artery and the ratio of balloon catheter diameter to arterial lumen.^[27]^ Although the invasive operation of IABP makes its complications difficult to avoid, it is helpful for the patients with AMI complicated with cardiogenic shock to tide over the difficulties by taking relevant measures to prevent and manage the complications effectively.

## 3. Diagnostic criteria of AMI and definition of cardiogenic shock

### 3.1. The AMI diagnostic criteria of WHO

The duration of chest pain is > 30 minutes and cannot be relieved after rest or nitroglycerin; the changes of dynamic electrocardiogram with or without abnormal Q wave; the dynamic changes of serum troponin and myocardial enzymes.

### 3.2. Definition of cardiogenic shock

It refers to the clinical manifestations of systolic blood pressure < 90 mmHg lasting at least 1 hour, accompanied by extremely low cardiac output, severe systemic circulatory failure, tissue hypoperfusion, such as persistent hypotension, chills in the extremities, oliguria, irritability, disturbance of consciousness, peripheral cyanosis, etc, as well as chest X-ray signs of pulmonary congestion.

## 4. Application of IABP in AMI complicated with cardiogenic shock

### 4.1. The application of IABP benefits AMI patients with cardiogenic shock

The application of IABP in cardiogenic shock has been controversial. After the neutral results of IABP- SHOCK II test, the international guidelines downgraded its use, but there is still a lack of clinical data on the long-term benefits of IABP in patients with AMI with cardiogenic shock. Moreover, there is only limited evidence for the long-term efficacy of IABP in patients with cardiogenic shock.^[31]^ Studies have shown that for high-risk patients undergoing coronary artery surgery, including acute myocardial infarction with cardiogenic shock, compared with intraoperative IABP, preoperative patients have significant benefits, and there are no vascular problems after IABP implantation.^[32]^ In 1 study, 84 patients with AMI complicated with coronary syndrome treated by emergency percutaneous coronary intervention were randomly divided into control group (n = 42) and observation group (n = 42). IABP was performed again in the control group after the internal medicine treatment was ineffective, and IABP was performed in the observation group before operation. The final results showed that early application of IABP after emergency percutaneous coronary intervention (PCI) in patients with AMI complicated with coronary syndrome could improve hemodynamics, increase the success rate of operation, reduce perioperative mortality, and did not increase the incidence of IABP complications, but had little effect on long-term survival and cardiac function indexes.^[33]^ In contrast, another subanalysis of the IABP- SHOCK II test showed that the timing of IABP implantation had no effect on the outcome of AMI patients with cardiogenic shock.^[34]^ Although IABP has been widely used in AMI, there are few clinical trials on the characteristics of the deceased. One study explored the clinical characteristics and risk factors of hospital death in patients with AMI supported by IABP. The clinical data of 572 patients with AMI who received IABP within 72 hours from July 2005 to July 2013 were analyzed retrospectively. The evolution and clinical characteristics of risk factors of in-hospital death in 81 dead patients and 81 surviving patients were compared. Finally, it is concluded that IABP support combined with revascularization may be more effective in patients with AMI with impaired hemodynamics, and patients whose hemodynamic parameters are significantly responsive to IABP may benefit from IABP to improve hospital survival.^[35]^

In an IABP randomized trial of cardiogenic shock patients, mechanical support had a slight effect on the reduction of acute physiology and chronic health evaluation II (APACHEII) score as a marker of disease severity, improvement of cardiac index, reduction of inflammatory state, or reduction of BNP biomarker status compared with drug treatment alone. However, the limitation of the trial is that it excludes any definitive conclusions and requires a larger prospective, randomized, multicenter trial with mortality as the main end point.^[36]^ A multicenter randomized clinical trial shows that careful use of prophylactic aortic balloon counterpulsation can prevent reocclusion of infarct-related arteries and improve overall clinical outcomes in patients undergoing acute cardiac catheterization during myocardial infarction.^[37]^ A study shows that IABP provides temporary hemodynamic and clinical improvements for most patients with cardiogenic shock. For hospitals without interventional cardiac catheterization laboratories, the use of IABP through thrombolytic therapy to increase patency or stabilize patients in order to transfer patients to hospitals with angioplasty services seems to be a promising strategy.^[5]^ A retrospective real-world cohort study found that 1028 patients with cardiogenic shock were evaluated, of whom 384 received IABP and 644 did not receive IABP. The hospital mortality rate of patients receiving IABP was significantly lower. Analysis of secondary endpoints found that the use of IABP was associated with a significant reduction in mortality within 1 year. After propensity score matching, the hospital mortality rate in the IABP group was still significantly lower than that in the control group, but these results need to be confirmed in future studies because of the complexity of the pathophysiological mechanism of cardiogenic shock and the diversity of existing evidence. A domestic study shows that for patients with AMI complicated with cardiogenic shock, IABP implantation can improve the short-term prognosis.^[38]^ IABP implantation before interventional diagnosis and treatment tends to improve survival, but it is not an independent protective factor for 28-day prognosis.^[39]^ An analysis based on propensity matching score shows that preoperative IABP implantation for high-risk left main disease can improve circulatory stability, reduce the incidence of intraoperative complications, and enable operators to deal with lesions more calmly.^[40]^ Another domestic study also shows that IABP combined with interventional therapy for critically ill AMI patients can reduce myocardial inflammatory injury, reduce cardiac load, improve cardiac function, reduce the incidence of cardiovascular adverse events and mortality, with high safety and effectiveness.^[41]^ In a 2-year long-term follow-up study, IABP had no adverse effect on the overall prognosis of patients with PCI after using propensity score to match baseline data. A domestic study evaluated the short-term and long-term prognostic effects of IABP before interventional therapy in patients with acute ST segment elevation myocardial infarction (STEMI) over 80 years old. The results showed that the incidence of major adverse cardiovascular events 1 month after PCI implantation in STEMI patients over 80 years old was significantly lower than that of conventional PCI, but there was no significant difference in short-term and long-term survival rates, and there was no significant predictive effect on each end event.^[42]^

### 4.2. The use of IABP cannot benefit patients with AMI complicated with cardiogenic shock

In current international guidelines, the recommendation for IABP in patients with AMI with cardiogenic shock is downregulated based on registration data. In the largest randomized trial (IABP-SHOCKII), IABP support did not reduce 30-day mortality compared with the control group.^[43]^ IABP-SHOCKII is a multicenter, randomized, open trial in which 600 patients with acute myocardial infarction with cardiogenic shock who underwent early revascularization between 2009 and 2012 were randomly divided into IABP group and control group. After the initial random grouping, long-term follow-up was performed for 6 months, 12 months and 6.2 years (quartile range 5.6–6.7), respectively. The final results showed that IABP could not reduce 12-month all-cause mortality.^[43]^ During the 6-year follow-up period, it had no effect on the overall mortality, which remained high, and 2/3 of patients with cardiogenic shock died despite simultaneous revascularization surgery.^[31]^ A meta-analysis of 7 randomized controlled trials showed that 4 of the trials compared IABP with standard treatment, and 3 trials compared it with other percutaneous left ventricular assist devices. A total of 790 patients with AMI and cardiogenic shock were included, 406 patients were treated with IABP and 384 patients served as control group. The hazard ratio of 30-day all-cause mortality was 0.95 (95% confidence interval 0.76–1.19), and no evidence of survival benefits was provided. The authors conclude that existing evidence suggests that IABP may have beneficial effects on some hemodynamic parameters, however, this does not bring survival benefits, so there is no convincing random data to support the use of IABP in infarct cardiogenic shock.^[44]^ A study evaluated the clinical characteristics and results of patients with cardiogenic shock treated with IABP before and after the European Society of Cardiology reduced the use of IABP from Class I to IIb in 2012. This is a prospective observational national survey conducted every 2 years. Of a total of 15,200 patients with acute coronary syndrome (ACS), 524 were identified as AMI with cardiogenic shock. These groups were further subdivided according to whether IABP was implanted before or after the recommended changes in the guidelines, and the final results showed that IABP as a treatment for cardiogenic shock decreased over time since the guidelines were revised. After the guidelines were changed, the use of IABP was limited to patients at high risk, severe impairment, and hemodynamic deterioration, thus limiting beneficial results.^[45]^ A real-world study shows that mortality is still high in patients with ACS who have cardiogenic shock and need resuscitation or mechanical ventilation despite the use of IABP. For ACS patients with non-cardiogenic shock, especially non-STE-ACS patients, IABP seems to be safe and often beneficial.^[46]^ In a randomized prospective trial, additional IABP therapy did not result in significant hemodynamic improvement in cardiogenic shock patients after PCI compared with drug therapy alone. Therefore, the application and recommendation of IABP in cardiogenic shock is still unclear.^[47]^ An analysis shows that the mortality rate of IABP is no better than that of non-IABP in patients with cardiogenic shock who receive early revascularization, that is, routine use of IABP does not increase any death benefits in patients with STEMI with cardiogenic shock.^[48]^ A domestic tendency matching analysis did not show that there was a statistically significant difference in intraoperative mortality, postoperative bleeding and the incidence of complications such as stent thrombosis between the IABP group and the non-IABP group, while the in-hospital mortality of the IABP group was significantly higher than that of the control group, which may be related to the more serious condition of the IABP group. The final conclusion shows that IABP cannot reduce the in-hospital mortality of STEMI patients receiving emergency interventional therapy.

## 5. Conclusion

At present, IABP has been widely used in clinic, and it is still a safe operation. For patients with AMI with cardiogenic shock, IABP should be used as soon as possible to improve the hemodynamic effects of patients; secondly, infarct-related arteries should be rapidly opened in combination with PCI; again, reasonable combination of drug therapy should be used to shorten the time for patients to use IABP and reduce the occurrence of complications. Despite the use of IABP, patients with cardiogenic shock still have a high mortality rate, so death risk assessment is particularly important. Some studies have shown that Intermountain Risk Score (IMRS) can evaluate short-term and long-term mortality in patients with cardiogenic shock and further predict short-term and long-term prognosis.^[49]^ This score can also be used in patients with IABP since the patient group is very similar. To sum up, although IABP plays a very important role in improving left ventricular ejection, increasing coronary perfusion pressure and reducing myocardial oxygen consumption, whether it can reduce the mortality of these patients remains to be tested in larger prospective randomized controlled clinical trials.

## Author contributions

**Writing – original draft:** Mengxian Li.

**Writing – review & editing:** Liqun Hu, Lei Li.
